# Time to Hang Up the Gloves: A Scoping Review of Evidence on Non‐Sterile Glove Use During Intravenous Antimicrobial Preparation and Administration

**DOI:** 10.1111/jan.70197

**Published:** 2025-09-25

**Authors:** Natasya Raja Azlan, Debbie Massey, Lesley Andrew, Amanda Towell‐Barnard, Seng Giap Marcus Ang, Carol Crevacore, Martina Costello, Aaron Alejandro, Weiting Liu, Naila Zaman, Peta‐Anne Zimmerman

**Affiliations:** ^1^ School of Nursing and Midwifery Edith Cowan University Joondalup Western Australia Australia; ^2^ Griffith University Southport Queensland Australia; ^3^ Centre for Nursing Research Sir Charles Gairdner Osborne Park Health Care Group Nedlands Western Australia Australia; ^4^ Clinical Services Hollywood Private Hospital Nedlands Western Australia Australia; ^5^ Infection Control Department Gold Coast Health Southport Queensland Australia; ^6^ Collaborative for the Advancement of Infection Prevention and Control Griffith University Southport Queensland Australia

**Keywords:** evidence‐based practice, hand hygiene, infection prevention and control, intravenous antimicrobials, non‐sterile glove use, patient safety, sustainable healthcare

## Abstract

**Aims:**

To systematically summarise evidence related to the use of non‐sterile gloves when preparing and administering intravenous antimicrobials.

**Design:**

Scoping review.

**Methods:**

A rigorous scoping review was undertaken following Arksey and O'Malley's (2005) framework and the modified Preferred Reporting Items for Systematic Reviews and Meta‐analyses extension for scoping review guidelines (2018). Five databases and grey literature were included in the search. Literature published between 2009 and 2024 was included.

**Data Sources:**

Five databases (Medline, CINAHL, EMBASE, Scopus and Web of Science) and the grey literature were searched in February 2024.

**Results:**

Three studies were included; however, none directly addressed correct non‐sterile glove use during intravenous antimicrobial preparation or administration in clinical practice.

**Conclusion:**

We found no evidence to support the use of non‐sterile gloves in intravenous antimicrobial preparation. There is an urgent need for rigorous research to inform the development of clear guidelines on non‐sterile glove use to underpin evidence‐based decision‐making in nursing and other health professional education, improve patient outcomes, reduce healthcare costs and promote environmental sustainability in healthcare.

**Implications:**

Inappropriate use of non‐sterile gloves for preparing and administering intravenous antimicrobials hinders correct hand hygiene practices and increases healthcare‐associated infections, healthcare costs and waste.

**Impact:**

A critical gap in the existing evidence was a key finding of this review, highlighting the urgency for evidence‐based guidelines to improve patient safety outcomes, reduce healthcare costs and promote environmental sustainability in healthcare.

**Reporting Method:**

This scoping review adhered to the relevant EQUATOR guidelines and Preferred Reporting Items for Systematic Reviews and Meta‐Analyses extension for Scoping Reviews (PRISMA‐ScR) reporting checklist.

**Patient of Public Contribution:**

This study did not include patient or public involvement in its design, conduct or reporting.

**Trial and Protocol Registration:**

The protocol was registered on Open Science Framework (https://doi.org/10.17605/OSF.IO/QY4J2).

## 1 Introduction

Inappropriate practice of non‐sterile glove use inhibits correct hand hygiene (Loveday et al. 2014), increases healthcare‐associated infections and incurs unnecessary financial costs (Wilson et al. 2015). The inappropriate use of non‐sterile gloves, including continued wearing when removal is required, is prevalent in nursing practice (Lindberg et al. 2020). No guidelines address non‐sterile glove use during intravenous antimicrobial preparation or administration, despite this being a common nursing practice. While general guidelines on non‐sterile glove use exist, they do not provide direction for this specific context (Centers for Disease Control and Prevention 2016; World Health Organization 2017). As such, no evidence‐based protocols exist to guide nurses and other health professionals in this practice. In this scoping review, we aimed to summarise available evidence on the correct use of non‐sterile gloves during preparation and administration of IV antimicrobials.

Hand hygiene is considered the most important aspect of infection prevention and control (IPC) (Mathur 2011). Effective hand hygiene significantly diminishes healthcare‐associated infections and consequently reduces antimicrobial resistance (Loveday et al. 2014). Despite its critical importance, poor compliance with hand hygiene protocols is evident among healthcare workers globally (Wilson et al. 2015).

Non‐sterile glove use became widespread in the late 1980s with the introduction of universal precautions, in response to the HIV epidemic, and has since become part of standard precautions internationally (Centers for Disease Control (US) 1988; Garner 1996; Centers for Disease Control and Prevention 2016; Loveday et al. 2014; Curran 2015). The use of non‐sterile gloves among nurses has increased in recent years and this practice is often taught to undergraduate student nurses during clinical skills education (Bate 2024; Loveday et al. 2014). This practice may have been influenced by researchers stating non‐sterile glove use reduces the risk of pathogen transmission in the healthcare setting (Baloh et al. 2019; Loveday et al. 2014; Wilson et al. 2015), transient colonisation of hands by micro‐organisms (Baloh et al. 2019; Bate 2024) and skin exposure to harmful and irritant chemicals (Hamnerius et al. 2018; Sadule‐Rios and Aguilera 2017).

Within our educational and clinical nursing practice, we have also observed the common use of non‐sterile gloves during IV antimicrobial preparation and administration, and the lack of a clear rationale for this. More broadly, published studies suggest unwarranted use of non‐sterile gloves occurs in up to 50% of patient interactions (Loveday et al. 2014; Wilson et al. 2015).

Consequences of incorrect non‐sterile glove use include poorer patient outcomes, raised financial costs and increased environmental harm. The reduced likelihood of hand hygiene compliance associated with non‐sterile glove use increases the potential for pathogen transmission and increases the risk of infection, long‐term complications and mortality (Fuller et al. 2011; Wilson et al. 2015). Globally, it is estimated that seven in every 100 patients in high‐income countries and 15 in low‐ and middle‐income countries will acquire a healthcare‐associated infection during their hospital admission (World Health Organization 2022). Neglecting to change non‐sterile gloves between patients and procedures, and not performing hand hygiene, is the most common reason for this cross‐contamination (Lindberg et al. 2020; Loveday et al. 2014).

The environmental impact of incorrect use of non‐sterile gloves in healthcare is wide ranging (Hambraeus 2006). Excessive non‐sterile glove usage contributes to increased medical waste and environmental pollution, a problem that has been magnified by the COVID‐19 pandemic (Andeobu et al. 2022; Jędruchniewicz et al. 2021; Loveday et al. 2014; Wilson et al. 2015). All steps in producing non‐sterile gloves and their eventual disposal impact the environment. Non‐sterile gloves are typically made from materials such as latex, nitrile or vinyl, which are not biodegradable (Jamal et al. 2021). The production of non‐sterile gloves consumes fossil fuels, water and energy (Jamal et al. 2021). Similarly, disposal of non‐sterile gloves impacts the environment, with most non‐sterile gloves being incinerated, which impacts air quality and releases deadly chemicals into the environment (Andeobu et al. 2022; Biederman et al. 2021). If non‐sterile gloves are disposed of into landfill, leachable substances such as microparticles and heavy metals contaminate drinking water and the food chain, which may cause ill health effects in humans and animals (Jędruchniewicz et al. 2021). By using non‐sterile gloves inefficiently or incorrectly, healthcare facilities are contributing to unnecessary waste and environmental harm (Andeobu et al. 2022).

The financial cost of unnecessary non‐sterile glove use extends past the cost of the resource. The increased risk of pathogen transmission can also be significant, through heightened treatment costs, extended hospital stays and greater antimicrobial resistance (Godijk et al. 2022; Lindberg et al. 2020). The World Health Organization (2022) reports that improved hand hygiene, inclusive of appropriate non‐sterile glove use, could save approximately $16.50 USD in reduced healthcare costs for each dollar invested in an IPC programme.

The lack of reliable evidence and the wide‐ranging consequences of incorrect non‐sterile glove use underpin the importance of this scoping review. The findings will inform future clinical practice guidelines, policies and processes, as well as nursing and other health professional education curricula.

## 2 The Review

### 2.1 Aim

The aim of this scoping review is to summarise the available evidence on non‐sterile glove use during preparation and administration of IV antimicrobials.

### 2.2 Methods

A scoping review approach was chosen because of its ability to produce a comprehensive and broad analysis of literature in an under‐examined area of research (Mays et al. 2004). We followed the modified Preferred Reporting Items for Systematic Reviews and Meta‐analyses extension for scoping review (PRISMA‐ScR) guidelines (Tricco et al. 2018) and the Arksey and O'Malley (2005) scoping review framework. We began by selecting a team of healthcare professionals consisting of nursing researchers, nurse educators and an expert clinician/educator/researcher in IPC. We followed a structured framework to ensure transparency in the methodological and analytical decisions undertaken throughout the review. The framework included six steps: (i) identifying a question, (ii) identifying relevant studies, (iii) study selection, (iv) data charting and collating, (v) summarising and (vi) reporting the results (Arksey and O'Malley 2005). The protocol for the scoping review was developed in collaboration between all authors and registered with Open Science Framework (https://doi.org/10.17605/OSF.IO/QY4J2).

### 2.3 Step 1: Identifying a Question

We aimed to systematically map current literature and identify available evidence about correct use of non‐sterile gloves during intravenous antimicrobial preparation and administration. For this research, antimicrobials include all agents but predominantly antibiotics. Scoping reviews do not use structured questions as they look to explore the breadth of research available on a topic (Crilly et al. 2010). The review was guided by the question, ‘What is the evidence supporting non‐sterile gloves use during IV antimicrobial preparation and administration?’

### 2.4 Step 2: Identifying Relevant Studies

An initial search was conducted in January 2024 using Medline and Cumulative Index for Nursing and Allied Health (CINAHL) databases to identify relevant keywords and index terms. The identified keywords and index terms were then undertaken in other databases including Embase, Web of Science and Scopus. The search was also extended to the grey literature including government‐related health websites such as the Australian Commission on Safety and Quality in Health Care (ACSQHC), New South Wales Clinical Excellence Commission (NSWCEC), New Zealand Ministry of Health (NZMOH) and World Health Organization, as well as other relevant websites such as Aseptic Non‐Touch Technique (ANTT). Reference lists of all included studies were examined to identify further potentially relevant studies.

The keywords and index terms were developed based on three main concepts: glove, antibiotics, antimicrobials and drug administration and reviewed by the discipline librarian. In addition, Boolean operators ‘AND’ and ‘OR’ and truncation ‘*’ were used for refining the search. The full search strategy is reported in Table 1.

**TABLE 1:** Search strategy and results across databases.

No	Search	Medline	CINAHL	Embase	Scopus	Web of Science
1	Glove*	22,195	7744	31,338	33,366	38,310
2	Gloves, surgical	3645	351	3242	5776	2242
3	Gloves, protective	3002	291	3675	6204	2362
4	MH “gloves, surgical” (use MH “gloves”)^a^	3080	3258			
5	MH “gloves, protective”	2206				
6	Antibiotic*	494,560	98,434	1,022,181	1,026,686	475,283
7	Antimicrobial*	302,271	28,884	404,971	408,474	420,905
8	Anti‐bacterial* (use antibacterial*)^a^	412,798	8312	191,599	325,621	218,165
9	Anti‐bacterial agent*(use antibacterial agent*)^a^	409,274	975	114,449	306,670	59,349
10	Antifungal*	100,364	10,980	147,800	174,476	109,080
11	Antifungal agent*	73,476	7907	103,628	122,598	36,877
12	Antiviral*	181,973	26,101	243,855	235,872	155,804
13	Antiviral agent*	107,691	20,648	107,616	150,557	30,054
14	Antimicrobial stewardship*	10,019	3497	17,667	13,069	11,558
15	MH “Anti‐bacterial agents”	408,624				
16	MH “antifungal agents”	68,349	7319			
17	MH “antiviral agents”	101,545	20,085			
18	MH “antimicrobial stewardship”	3491	1855			
19	Drug* admin*	284,965	88,460	4,098,999	2,151,059	392,611
20	Med* admin*	85,908	35,613	4,965,322	944,619	1,182,747
21	Drug* prep*	17,455	1561	715,190	665,474	247,894
22	Med* prep*	37,539	6318	1,759,386	576,963	1,060,952
23	Pharm* prep*	81,850	1839	437,874	219,453	331,298
24	MH “pharmaceutical preparations”	69,211				
25	1 OR 2 OR 3 OR 4 OR 5	22,195	7744	31,338	33,366	38,310
26	6 OR … OR 15…18	1,090,696	149,903	1,605,387	1,670,751	1,081,473
27	19 OR …OR 24	484,225	127,794	6,490,834	3,439,779	2,428,975
28	25 AND 26 AND 27	33	6	687	202	143
29	Limit to 2009–Present	20	3	514	111	109

*Note:* * indicates truncation that was used in the database searches, retrieving all word variants beginning with the same root.

Abbreviation: CINAHL = Cumulative Index for Nursing and Allied Health.

^a^ For CINAHL, web of science, embase.

The scoping review included all primary research studies published between 2009 and 2024 reporting on non‐sterile glove use for the preparation and administration of IV antimicrobials and involving nurses. Studies published before 2009 were excluded to align with the World Health Organization's 2009 glove use guidelines, ensuring the evidence reflects current clinical practice. Articles that were not published in English, conference abstracts, reviews, reports and guidelines, and those that did not involve nursing research or practice were excluded.

### 2.5 Step 3: Study Selection

All citations identified in the search were imported to Endnote 20.1 (Clarivate Analytics). Duplicate citations were removed after being uploaded to the web‐based bibliographic manager—COVIDENCE, for independent screening of titles, abstracts and full‐text review according to inclusion criteria. Titles and abstracts were reviewed independently and concurrently for potential inclusion by two researchers (P.Z. and W.L.). Potentially relevant sources were retrieved in full‐text form and imported to COVIDENCE. Full‐text sources were independently reviewed by two researchers (P.Z. and W.L.) against the inclusion criteria. Any disagreement between the two researchers at the full text stage was resolved through discussion by the inclusion of a third reviewer (A.T.B.).

### 2.6 Step 4: Data Charting and Collating

The data extracted included the first author, year, country, aims, study design, methods, population, main findings and protocol for glove use. Cochrane (2023) recommends more than one person extract data to minimise errors and potential bias. In our scoping review, data were extracted from included sources by two independent reviewers (P.Z. and M.C.) using the data extraction tool developed by the research team. The data extraction process was then reviewed by a third researcher (C.C.) to ensure consensus of the accurate representation of included sources.

### 2.7 Step 5: Summarising and Reporting Results

Details of included sources are presented in subsequent sections.

## 3 Results

### 3.1 Search Results

**FIGURE 1:**
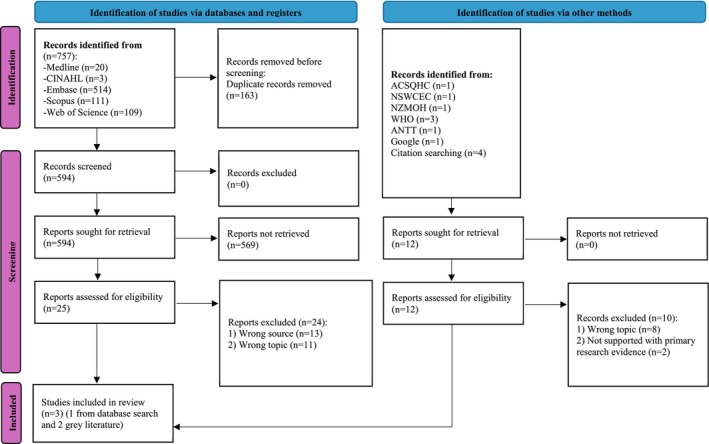
PRISMA flow diagram. ACSQHC = Australian Commission on Safety and Quality in Health Care, ANTT = Aseptic Non‐Touch Technique, CINAHL = Cumulative Index for Nursing and Allied Health, NSWCEC = New South Wales Clinical Excellence Commission, NZMOH = New Zealand Ministry of Health, WHO = World Health Organization.

The search generated 757 records, including Medline (*n* = 20), CINAHL (*n* = 3), EMBASE (*n* = 514), Scopus (*n* = 111) and Web of Science (*n* = 109). After removing 163 duplicates, the remaining 594 articles were screened using the inclusion and exclusion criteria against the title and abstract. From this, 25 articles were selected for full‐text review. The reference lists of these 25 articles were examined for further potential sources of evidence, resulting in no selection of additional articles. Of the 25 studies, 24 were excluded based on the reasons listed in Table 2. Another two studies were identified from the grey literature and included in this review, resulting in three studies overall. No studies directly provided primary evidence on non‐sterile glove use during intravenous antimicrobial preparation or administration, underscoring a critical evidence gap that warrants urgent investigation. The results of the search and the study inclusion process are presented in Figure 1 and the outcome of the grey literature search is presented in Table 3.

**TABLE 2:** Reason for exclusion of articles from database search.

No	First author and date	Title	Reason for exclusion
1	Martin et al. (2010)	Heightened infection‐control practices are associated with significantly lower infection rates in office‐based Mohs surgery	Wrong topic—does not involve glove use
2	Morgaonkar et al. (2016)	Intravenous line practices in neonates during neonatal intensive care unit (NICU) stay at tertiary care center in rural Gujrat, India	Wrong source—conference abstract
3	Singh et al. (2019)	Practices of nurses regarding handling of central line catheter at a tertiary care center	Wrong source—conference abstract
4	Ory et al. (2021)	Impact of antibiotic stewardship and implementation of infection control on prevalence of surgical site infections in a large tertiary‐care hospital in Haiphong city	Wrong topic—does not involve glove use
5	Faller et al. (2020)	How Standard Are Standard Precautions? Knowledge and Attitudes Towards Standard Precautions at an Academic Medical Center	Wrong source—conference abstract
6	Huang et al. (2017)	Aseptic technique maintains sterility of antibiotic‐loaded peritoneal dialysis fluid	Wrong source—conference abstract
7	Mahé et al. (2021)	A need to improve the monitoring of adverse effect after occupational exposure	Wrong source—conference abstract
8	Al‐Sayaghi et al. (2011)	Management of central venous catheters at the intensive care units in Yemen. Survey of practices	Wrong topic—does not involve glove use
9	Oburah et al. (2023)	Strategies on safe handling and disposal of cytotoxic products in preventing infection and improving infection control among health care works in MTRH‐Kenya	Wrong source—conference abstract
10	Pinheiro et al. (2018)	Occupational allergic contact dermatitis caused by antibiotics in healthcare workers—relationship with non‐immediate drug eruptions	Wrong topic—does not involve glove use
11	Harbarth (2013)	Healthcare‐associated infections: Think globally, act locally	Wrong source—conference abstract
12	Bish et al. (2014)	A socio‐technical, Probabilistic risk assessment model for surgical site infections in ambulatory surgery centers	Wrong topic—does not involve glove use
13	Elshami et al. (2017)	Practices of injections safety: A clinical audit at al‐shifaa hospital in the Gaza‐Strip	Wrong source—conference abstract
14	Galanis et al. (2021)	Healthcare professionals' knowledge and practices towards hospital infections in surgical clinics	Wrong source—conference abstract
15	Westwood et al. (2023)	Time for change: compliance with RCS green theatre checklist‐facilitators and barriers on the journey to net zero	Wrong topic—does not involve glove use
16	Brahimi et al. (2019)	Audit on good practices related to peripheral venous catheters in January 2019	Wrong source—conference abstract
17	Ababneh et al. (2022)	Isolation of extensively drug resistant *Acinetobacter Baumannii* from environmental surfaces inside intensive care units	Wrong topic—does not involve glove use
18	Pieri et al. (2010)	Biological monitoring of nurses exposed to doxorubicin and epirubicin by a validated liquid chromatography/fluorescence detection method	Wrong topic—does not involve glove use
19	Forrester et al. (2017)	Developing operating system process maps for surgical infection prevention: A tool to improve perioperative standards in low‐and middle‐income countries	Wrong source—conference abstract
20	Chen et al. (2017)	Investigation of orthopaedic arthroscopic surgery of *Staphylococcus aureus* infection outbreak	Wrong source—conference abstract
21	Zaiac et al. (2016)	Microbial contamination of disposable nitrile gloves: A prospective, randomised pilot study of a novel sterilisation technique for use in Mohs micrographic surgery	Wrong source—conference abstract
22	Kim et al. (2011)	Effect of routine sterile gloving on contamination rates in blood culture: A cluster randomised trial	Wrong topic—does not involve antibiotics administration
23	Giard et al. (2013)	Results of the French national audit on standard precautions	Wrong topic—does not involve antibiotics administration
24	Suvikas‐Peltonen et al. (2017)	Auditing safety of compounding and reconstituting of intravenous medicines on Hospital Wards in Finland	Wrong topic—does not include antibiotic administration by nurses

**TABLE 3:** Summary of grey literature search.

Source	Article information	Decision
ACSQHC	Therapeutic Guidelines. (2023). “Antibiotic Prescribing in Primary Care: Therapeutic Guidelines Summary Table 2023.” https://www.safetyandquality.gov.au/sites/default/files/2023‐05/gpsummary_v15.pdf	Excluded—Wrong topic—does not involve glove use
NSWCEC	Clinical Excellence Commission. (2020). “Infection Prevention and Control Practice Handbook.” https://www.cec.health.nsw.gov.au/__data/assets/pdf_file/0010/383239/IPC‐Practice‐Handbook‐2020.PDF	Excluded—Wrong topic—does not include antibiotic administration
NZMOH	Ministry of Health. (2019). “Medication Guidelines for the Home and Community Support Services Sector.” https://www.health.govt.nz/system/files/documents/publications/medication‐guidelines‐home‐community‐support‐services‐sector‐may19.pdf	Excluded—Wrong topic—does not involve glove use
WHO	World Health Organization. (2009). “WHO Guidelines on Hand Hygiene in Health Care First Global Patient Safety Challenge Clean Care is Safer Care.” https://iris.who.int/bitstream/handle/10665/44102/9789241597906_eng.pdf?sequence=1	Excluded—Wrong topic—does not include antibiotic administration
WHO	World Health Organization (2009). “Glove Use Information Leaflet.” Revised August 2009. https://www.who.int/publications/m/item/glove‐use‐information‐leaflet‐(revised‐august‐2009)	Excluded—not supported with primary research evidence
WHO	World Health Organization (2010). “WHO Best Practices for Injections and Related Procedures Toolkit.” https://www.who.int/publications/i/item/9789241599252	Excluded—not supported with primary research evidence
ANTT	Aseptic Non‐Touch Technique. (n.d.). https://www.antt.org/antt‐procedures‐settings.html#anchor2	Excluded—Wrong topic—does not include antibiotic administration
Google	Dunn et al. (2019). “A Programme to Cut Inappropriate Use of Non‐Sterile Medical Gloves.” *Nursing Times*, 115(9), 18–20. https://emap‐moon‐prod.s3.amazonaws.com/wp‐content/uploads/sites/3/2019/08/190821‐A‐programme‐to‐cut‐inappropriate‐use‐of‐non‐sterile‐medical‐gloves.pdf	Included
Citation search	Loveday et al. (2014). Clinical Glove Use: Healthcare Workers' Actions and Perceptions. *Journal of Hospital Infection*, 86(2), 110–116. https://doi.org/10.1016/j.jhin.2013.11.003	Excluded—Wrong topic—does not include antibiotic administration
Citation search	Pinheiro, V., Pestana, C., Pinho, A., Antunes, I., & Gonçalo, M. (2018). “Occupational Allergic Contact Dermatitis Caused by Antibiotics in Healthcare Workers—Relationship With Non‐Immediate Drug Eruptions.” *Contact Dermatitis*, 78(4), 281–286. https://doi.org/10.1111/cod.12960	Excluded—Wrong topic—does not involve glove use
Citation search	Kim, J. E., Kim, S. H., Jin, H. J., Hwang, E. K., Kim, J. H., Ye, Y. M., & Park, H. S. (2012). “IgE Sensitization to Cephalosporins in Health Care Workers.” *Allergy, asthma & Immunology Research*, 4(2), 85–91. https://doi.org/10.4168/aair.2012.4.2.85	Excluded—Wrong topic—does not involve glove use
Citation search	Wilson et al. (2017). “Applying Human Factors and Ergonomics to the Misuse of Nonsterile Clinical Gloves in Acute Care.” *American Journal of Infection Control*, 45(7), 779–786. https://doi.org/10.1016/j.ajic.2017.02.019	Included

Abbreviations: ACSQHC = Australian Commission on Safety and Quality in Health Care, ANTT = Aseptic Non‐Touch Technique, NSWCEC = New South Wales Clinical Excellence Commission, NZMOH = New Zealand Ministry of Health, WHO = World Health Organization.

An optional and exploratory quality assessment was conducted using the Mixed Methods Appraisal Tool (MMAT) to contextualise the rigour of included studies (Hong et al. 2018). While quality appraisal is not a required component of scoping reviews under PRISMA‐ScR guidelines (Tricco et al. 2018) or Arksey and O'Malley's (2005) scoping review framework, this assessment was conducted to provide additional context for interpreting the findings. Other authors have also used quality appraisal tools in scoping reviews to improve the quality of the review (Cole et al. 2024; Milou et al. 2024). The MMAT enables methodological appraisal of five domains (justification, integration, interpretation, disagreements and adherence) across a variety of study methods, to provide a detailed presentation of the quality of included studies. Where all MMAT criteria are either absent or unable to be determined, the study was considered a low‐quality study. The results of the appraisal are presented in Table 4.

**TABLE 4:** Characteristics of included studies.

Author, year, country	Aims	Design	Methods	Population	Findings	Protocol for glove use	MMAT results
Dunn et al. (2019), United Kingdom	To create an educational awareness program for staff around the use of gloves for standard infection‐control precautions and transmission‐based precautions	Quality improvement 12‐months program including educational package and risk assessment strategy for when to use gloves to prepare and administer intravenous medication using the WHO glove use pyramid	Impact measured by monitoring volume and cost of monthly gloves ordered and weekly associated waste, number of staff presenting to occupational health with skin issues, and hand‐hygiene compliance from infection control audits	Practice educators and central venous access, occupational health (OH), dialysis, pharmacy and total parental nutrition teams	After the program launch, non‐sterile glove orders fell by 3.7 million to 7.4 million; this resulted in savings of more than £90,000, number of non‐sterile gloves used decreased by 30% reducing the amount of waste by around 18 tonnes. No rise in new attendances of staff presenting to occupational health	Gloves are only needed when administering medication such as eye or nose drops, where there is a risk of coming into contact with body fluids or a mucous membrane, therapeutically active creams, liquid hormones and cytotoxic drugs	Criteria 3.3 and 3.4 for non‐randomised studies not met
Antunes et al. (2011), Portugal	Not stated	Case study	Patch tests with standard and gloves series	32 year old nurse with 1.5‐year history of hand dermatitis and 2 months history of palpebral eczema	Hand eczema specifically contact sensitisation to first, second and third generation cephalosporins	—	Criteria 4.2, 4.4 and 4.5 for quantitative descriptive studies were not applicable
Wilson et al. (2017), United Kingdom	To examine behaviour and attitudes of nonsterile glove use to inform strategies for reducing inappropriate nonsterile glove use and improve patient safety	Mixed method approach using observation and qualitative interviews	Observational audit Semi‐structured interviews	Healthcare workers from 2 acute care hospitals	178 out of 278 procedures performed involved the use of nonsterile glove with 59% (165 out of 278) were inappropriate. Risk of cross‐contamination occurred in 49% (87 out of 178) episodes. 26 healthcare workers interviewed; emotion and socialisation were 2 main themes influencing decisions to use nonsterile gloves	Nonsterile gloves used if the procedure involved potential or actual contact with blood and body fluids, mucous membranes, situations required by local policy	All criteria met for mixed methods studies

### 3.2 General Study Characteristics

Of the three studies, two were conducted in the United Kingdom (Dunn et al. 2019; Wilson et al. 2017), and one study was conducted in Portugal (Antunes et al. 2011). A full summary of study characteristics is presented in Table 4.

The three studies differed in their aims and methodologies. Dunn et al. (2019) aimed to develop and evaluate an educational program to create awareness of appropriate non‐sterile glove use. Although the study did not specifically focus on non‐sterile glove use in relation to the preparation and administration of IV antibiotics, the authors discussed that non‐sterile gloves are only used when administering medications such as eye or nose drops, where there is a risk of contact with body fluids or a mucous membrane, therapeutically active creams, liquid hormones and cytotoxic drugs. The quality improvement study applied a quantitative approach to measure the impact of the educational program, which included changes in the number of gloves ordered, the weight of non‐sterile gloves waste in the following year, the number of staff presenting to occupational health with skin issues and hand hygiene compliance. There was no further discussion on qualitative data collection or their findings in the article.

In contrast, Wilson et al. (2017) aimed to examine behaviours and attitudes regarding non‐sterile glove use to inform strategies for reducing inappropriate non‐sterile glove use and improve patient safety. The study applied a mixed‐methods study design incorporating observational audits and semi‐structured interviews. Wilson et al. (2017) identified from an audit that 165 (59%) of 278 procedures involving the use of non‐sterile gloves were inappropriate. Interviews with 26 healthcare workers, including 16 nurses, identified the influence of emotions, including fear, disgust, depersonalisation and ease of mind and socialisation, including policies and procedures, empathy and behaviour of peers influenced their decisions for using non‐sterile gloves.

Although Antunes et al. (2011) did not state an aim, they discussed a procedure to ascertain the cause of a nurse's hand eczema using a case study approach. A single nurse presenting with hand eczema underwent patch tests for allergy to a range of drugs they prepared for systematic administration, gloves and disinfectants. The results of the patch tests found positive results for medication preparation/administration and disinfectants.

### 3.3 Findings Specific to IV Antimicrobial Preparation and Administration and Non‐Sterile Gloves Use

Findings from both Wilson et al. (2017) and Dunn et al. (2019) were not specific to IV antimicrobial preparation or administration. Wilson et al. (2017) instead reported inappropriate non‐sterile glove use, finding this occurred in 59% of observed procedures (165 of 278 procedures) across the two study hospitals. No statistically significant differences were found between staff groups. The findings also described the following frequency of non‐sterile glove use: cleaning 13.3%; patient mobilisation 12.9%; linen handling 12.6%; IV device manipulation 10.1%; personal hygiene 7.6%; toileting 7.2%; handling equipment 7.2%; manipulation of non‐IV invasive device 5.8%; attending to patient 5.8%; no particular task 5%. The appropriateness or inappropriateness of these uses was not specified. Wilson et al. (2017) also reported findings relating to cross‐contaminations episodes, organising them against the ‘five moments of hand hygiene’ (World Health Organization 2009), this being moment 1 (before touching a patient) 21/37, moment 2 (before a procedure) 10/17, moment 3 (after a procedure or body fluid exposure risk) 15/27, moment 4 (after touching a patient) 30/53 and moment 5 (after touching a patient's surroundings) 12/21. Nurses were significantly less likely to decontaminate hands after glove removal than allied health professionals (48/111 vs. 1/14 *p* = 0.002). Qualitative findings from interviews with healthcare professionals on reasons behind non‐sterile glove use were organised into two themes. Theme one was ‘emotion’, with subthemes of ‘fear’, ‘disgust’, ‘psychological barrier’ and ‘ease of mind.’ Theme two was ‘socialisation’ with subthemes ‘professional’, ‘organisational’ and ‘empathetic’.

In contrast, the case study by Antunes et al. (2011) did discuss antibiotic preparation and administration; however, the study did not discuss this in terms of non‐sterile glove use. The study instead found a nurse's hand eczema was caused by the preparation of first‐, second‐ and third‐generation cephalosporins for systemic administration. Ceasing this role led to the resolution of skin eczema. Importantly, no discussion of the use of non‐sterile gloves in antibiotic preparation and administration was evident. Furthermore, the article did not discuss the possibility of wearing non‐sterile gloves in future antibiotic preparation as a potential solution to the development of eczema. The authors did not clarify if the administration of antibiotics specifically involved IV preparation and administration; however, it did mention ‘needle gushing’, suggesting this was an aspect of administration considered.

### 3.4 Cited Sources Used to Support Non‐Sterile Glove Use Rationales

Although Dunn et al. (2019) and Wilson et al. (2017) offered no evidence from their own research findings to support a rationale for non‐sterile glove use during IV antimicrobial preparation, both studies did offer a rationale for correct non‐sterile glove use. Dunn et al. (2019) stated non‐sterile gloves should only be used if exposure to bodily fluids or mucous membranes is ‘likely’ or ‘very likely’ and that they should not be used for IV antibiotic preparation and administration. While not discussing IV antimicrobial preparation at all, Wilson et al. (2017) also stated within their article introduction that the use of non‐sterile gloves was indicated where a risk of direct contact with blood or body fluids is anticipated.

**FIGURE 2:**
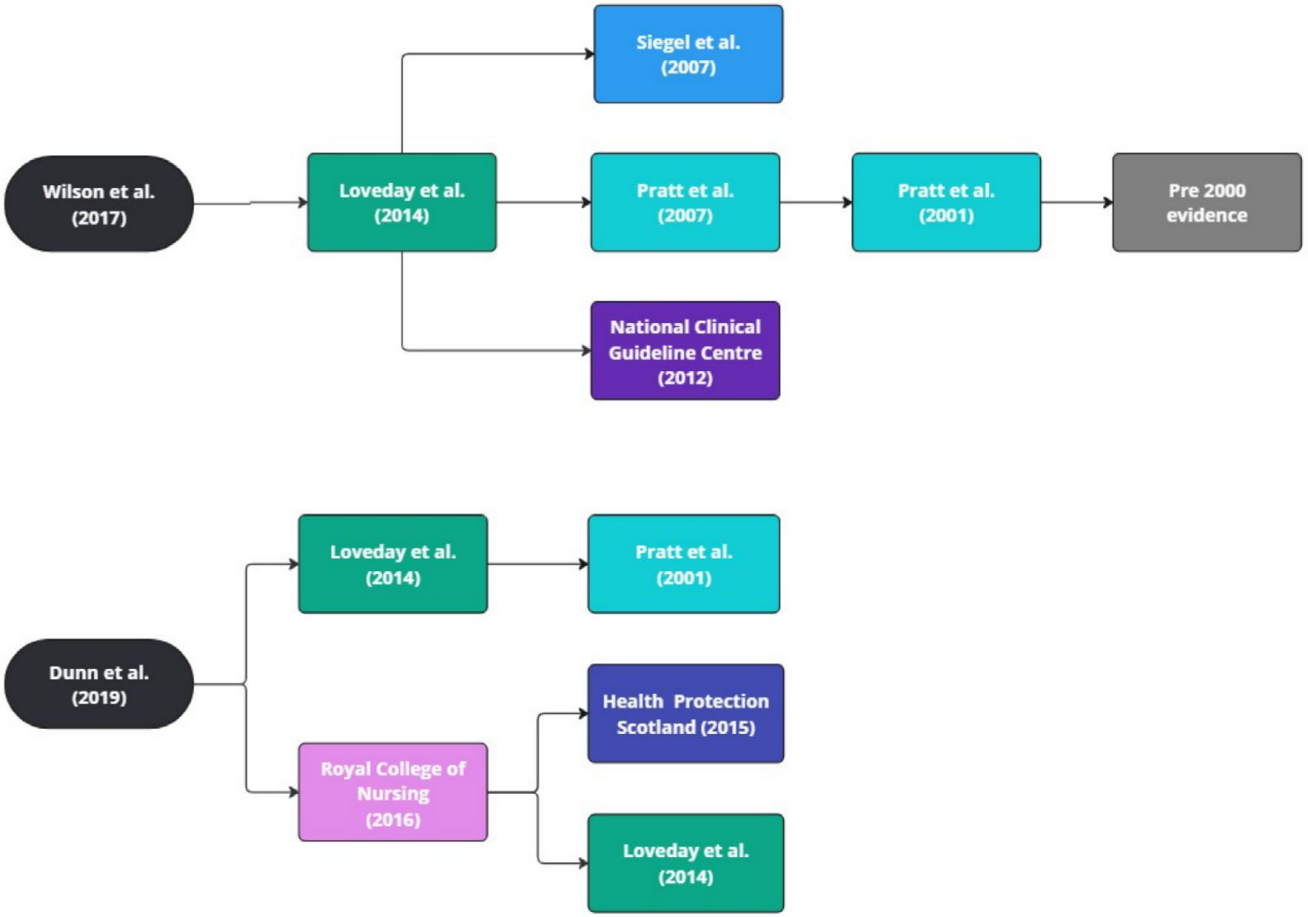
Flowcharts of source citations for non‐sterile glove use rationale.

Both Dunn et al. (2019) and Wilson et al. (2017) supported their non‐sterile glove use rationales with several cited sources. To find the original research evidence for non‐sterile glove use in IV antimicrobial preparation and administration, we examined the single cited source from Dunn et al. (2019), and the two cited by Wilson et al. (2017). As none of these three cited sources provided this evidence, the process was continued, and their cited sources were also examined. The examination of cited sources was ceased when publication dates predated 2000, with studies before this date deemed outdated. This process, while unusual in scoping review study analysis but important to the aim of the review, is summarised in Figure 2 and Table 5. The process resulted in the examination of eight articles, in addition to the three selected for review. None of these articles provided any research evidence to support or refute the use of non‐sterile gloves in IV antimicrobial preparation or administration.

**TABLE 5:** Sources of evidence cited in the selected studies related to non‐sterile glove use in general and in IV antimicrobial preparation and administration.

Article title, year	Rationale provided for non‐sterile glove use in general	Evidence cited	Rationale provided for non‐sterile glove use in IV antimicrobial preparation/administration	Evidence cited
*Selected study one*				
Wilson et al. (2017). Applying human factors and ergonomics to the misuse of nonsterile clinical gloves in acute care	Indicated where a risk of direct contact with blood or body fluids is anticipated	Loveday et al. (2014)	No	NA
Loveday et al. (2014) Clinical glove use: Healthcare workers actions and perceptions	Risk of direct contact with blood and other body fluids, and with a patient in an isolation room during a defined outbreak or infection and the use of hazardous chemicals such as disinfectant	Pratt et al. (2007) National Clinical Guideline Centre (UK) (2012) (cited in article as National Institute for Clinical Excellence, 2012) Siegel et al. (2007)	No	NA
Pratt et al. (2007). epic2: National Evidence‐Based Guidelines for Preventing Healthcare‐Associated Infections in NHS Hospitals in England	“Gloves (sterile/nonsterile not specified) must be worn for invasive procedures, contact with sterile sites, and non‐intact skin or mucous membranes, and all activities that have been assessed as carrying a risk of exposure to blood, body fluids, secretions and excretions; and when handling sharp or contaminated instruments” (p. S4)	Pratt et al. (2001)	No	NA
National Clinical Guideline Centre (UK) (2012) (cited in article as National Institute for Clinical Excellence, 2012). Infection: prevention and control of healthcare‐associated infections in primary and community care	“Gloves must be worn for invasive procedures, contact with sterile sites and non‐intact skin or mucous membranes, and all activities that have been assessed as carrying a risk of exposure to blood, body fluids, secretions or excretions, or to sharp or contaminated instruments.”(p. 35)	No cited evidence	No	NA
Siegel et al. (2007). Guideline for Isolation Precautions: Preventing Transmission of Infectious Agents in Health Care Settings	For touching blood, body fluids, secretions, excretions, contaminated items, mucous membranes and nonintact skin	None relevant	No	NA
Pratt et al. (2001). The epic project: developing national evidence‐based guidelines for preventing healthcare‐associated infections. Phase I: Guidelines for preventing hospital‐acquired infections	To protect hands from contamination with organic matter and microorganisms and to reduce the risks of transmission of microorganisms to both patients and staff	Pre‐2000 cited evidence	No	NA
*Selected study two*				
Dunn et al. (2019). A programme to cut inappropriate use of non‐sterile medical gloves	If exposure to bodily fluids or mucous membranes is likely or very likely	Loveday et al. (2014) Royal College of Nursing (2016)	No	NA
Loveday et al. (2014). epic3: national evidence‐based guidelines for preventing healthcare‐associated infections in NHS hospitals in England	To protect hands from contamination with organic matter and microorganisms; and to reduce the risk of cross‐transmission of microorganisms to staff and patients. Also, the use of some chemicals or medications	Pratt et al. (2001)	No	NA
Royal College of Nursing (2016). Standards for Infusion Therapy (currently under review)	“Practitioners performing procedures that result in the generation of aerosols, droplets or splashing of blood and/or body fluids should ensure that they are undertaking the appropriate transmission‐based precautions and using appropriate personal protective equipment including well‐fitting gloves” (p.19) “Gloves should be worn to protect hands from contamination from blood, body fluids, secretions and excretions and to reduce the risk of cross‐contamination to both patient and staff” (p.21)	Health Protection Scotland (2015) (no longer available online) Loveday et al. (2014)	Yes. “Gloves are not required for the preparation of antibiotic infusions to prevent exposure to the drug. Concerns regarding sensitivity to antibiotics should be discussed with local occupational health advisers” (p.21)	None cited

### 3.5 Quality of Evidence

The quality of the three selected studies was assessed using the Mixed Methods Appraisal Tool (MMAT) revealing variable approaches to and compliance with rigour and transparency (Table 4). Our initial observations suggest the included studies lacked rigour, and we sought to confirm these concerns through a structured and objective assessment process. Regardless of the quality of the research identified, all articles were included because of the dearth of available studies. Antunes et al.'s (2011) study, for example, was a single‐participant case study, limiting the reliability and validity of findings. The description of the study (letter) created discussion among the scoping review team about its relevance; however, because of the lack of studies and the fact that the manuscript was identified as research, we deemed its inclusion warranted.

It is worth noting that an examination of the three selected articles and the further eight articles cited revealed some similarities. The article by Loveday et al. (2014), for example, was very similar in aim, design and findings to the article by Wilson et al. (2017). Like Wilson et al. (2017), the Loveday et al. (2014) study explored the reasons behind the incorrect use of gloves, via observation and healthcare worker interviews across hospitals in England. In addition, an examination of members of the research teams across the three selected and eight further cited articles showed many were undertaken by the same researchers, notably Loveday et al. (2014) (in four studies), Wilson et al. (2017) (in four studies) and Pratt et al. (2001) (in three studies). While not uncommon in specialised research areas, this clustering of authorship underscores the limited pool of investigators currently contributing to this topic. These findings further suggest research in this area is limited, highlighting the need for broader engagement from the wider research community to diversify perspectives and expand the evidence base.

### 3.6 Supplementary Findings

While not directly answering the scoping review question, the included manuscripts provided evidence on reasons for incorrect non‐sterile glove use. From their interviews with 26 healthcare workers, Wilson et al. (2017) found non‐sterile glove use was commonly linked to incorrect perceptions of risk to self from handling IV antibiotics, related to current antibiotic allergy or fear of developing an allergy due to repeated skin exposure. There was no discussion of why these perceptions were incorrect or any analysis of this finding.

Dunn et al. (2019) discussed the reasons behind incorrect non‐sterile glove use. These included the misperception that they were needed for IV antibiotic preparation, to improve ‘cleanliness’, and a general lack of awareness that non‐sterile gloves may be contaminated and therefore lead to cross‐contamination. It was not clear, however, if these were findings from their current study or a previous IPC audit undertaken earlier in the hospital (and which was not cited).

## 4 Discussion

In this scoping review, we employed a rigorous and comprehensive approach to find evidence to answer the question: ‘What is the existing evidence for wearing non‐sterile gloves during antimicrobial preparation and administration?’ The lack of reliable or contemporary evidence means this question remains unanswered. The review also revealed the practice of citing previous sources of evidence on non‐sterile glove use more broadly, which, on deeper examination, provided no primary evidence themselves. The current ritualistic practice of non‐sterile gloves being worn for IV antimicrobial preparation and administration points to an important gap in knowledge and a lack of high‐quality research in this important clinical area. The analysis revealed that much of the existing research on non‐sterile glove use originates from a relatively small group of recurring investigators. While their expertise contributes important insights, this concentration may restrict the range of perspectives and methodological approaches applied to the topic. Diverse research teams may promote innovation, validate findings through replication and strengthen the reliability of evidence. Thus, contributions from a wider pool of researchers are important to generate additional robust, comprehensive and generalisable evidence to inform clinical practice and policy.

Two of the included studies in this review considered healthcare worker's skin health in their findings. Dunn et al. (2019) reported no rise in staff hand skin issues from discontinuing glove use when not indicated, although no specific mention of antibiotic preparation and administration were made with reference to skin issues. The case study by Antunes et al. (2011) found the preparation of antibiotics (cephalosporins) was associated with skin reactions in a single case study, and the cessation of this practice resolved the issue. No mention of the use of non‐sterile gloves was made. Neither therefore directly discussed the implications for skin health of non‐sterile glove use in IV antimicrobial preparation and administration. A limitation of these findings' ability to answer the scoping review question is that Dunn et al. (2019) did not mention practices involving IV antimicrobial preparation and administration, and Antunes et al. (2011) did not mention non‐sterile glove use. However, these findings reflect how skin health concerns may influence glove use in practice and highlight the lack of direct evidence specific to IV antimicrobial preparation.

Prior research in this area is mixed, with the two studies cited by Antunes et al. (2011) on the topic providing conflicting results. The first by Gielen and Goossens (2001), reported on 33 cases of occupational allergic contact dermatitis among health workers presenting to a Belgian hospital between 1980 and 2001. They found the most common causes of the contact dermatitis was allergy to antibiotics (most commonly penicillin and cefuroxime), causing 35 positive patch tests. Nurses were the most sensitised patients (26/33 presenting). IV antimicrobial preparation and administration was not specifically discussed (Gielen and Goossens 2001). Powdered substances were identified as causing a reaction through the airborne route as well as through crushing of drugs for preparation (Gielen and Goossens 2001). There is also continuing advice to avoid substance preparation by people with allergies (and the use of patch tests to determine causes of contact dermatitis), as well as the use of gloves and other personal protective equipment for IV antibiotic preparation as a ‘strategy of avoidance’ (Gielen and Goossens 2001).

Schnuch et al. (1998) undertook their study in Germany between 1992 and 1995 in 24 allergy departments and analysed patch tests of 31,849 patients, finding higher rates of sensitisation among healthcare workers than the public. The most common allergens for healthcare workers were rubber, thiomersal (vaccine preservative) and biocides (surface and instrument disinfectants). Schnuch et al. (1998) reported medicaments (penicillins and cephalosporins) ‘do not appear to be important in our study’ (p. 362) compared to other allergens (such as rubber in gloves and biocides used as disinfectants).

Wilson et al. (2017) identified that non‐sterile glove use was often driven by perceived personal risk when handling IV antibiotics, including concerns about allergic reactions. More broadly, the reasons for healthcare workers non‐sterile glove use in general in this study were found to be incorrect perception of risk of contamination driven by ‘fear and disgust’ of perceived pathogens ‘germs and dirt’ and the misconception that non‐sterile glove use was more hygienic than performing hand hygiene. Healthcare workers also reported being socialised into the practice of routinely wearing non‐sterile gloves, which was described as normal and unquestioned on the wards. Dunn et al. (2019) identified misconceptions about glove use for IV antibiotic preparation and a limited understanding of contamination risks as contributing factors to misuse.

A literature review exploring the correct use of both sterile and non‐sterile glove use published in 2023 for NHS Scotland reveals primary evidence on the subject and no specific legislative requirements for the use of gloves as personal protective equipment for IPC purposes (NHS National Services Scotland 2023). The review instead summarised expert opinion guidelines across the UK, Australia, the USA, India, the Republic of Ireland and by the World Health Organization on when non‐sterile gloves should be used. This expert opinion states non‐sterile glove use should be dependent on the risk of exposure to patients and healthcare workers to blood, body fluids, mucous membranes, skin breaks and hazardous chemicals—reflecting the guidelines cited in our three selected studies. The NHS Scotland review did not offer expert opinion specific to IV antibiotic preparation and administration. The authors of this review did recommend future research to offer evidence on the subject of correct non‐sterile glove use, a recommendation reiterated by Ricciardi and Cascini (2021) in the textbook of Patient Safety and Clinical Risk Management.

The quality of the research of the three selected articles in our scoping review further detracts from our confidence to identify clear evidence for the use of non‐sterile gloves in the preparation and administration of IV antimicrobials.

Our observations of inappropriate non‐sterile glove use among qualified and student nurses within Australia prompted the examination of health service and community policy and procedures as well as educational resources. Hospital and community policy, procedures and standard operating procedures available on the internet offer some advice (The Royal Hospital For Women 2017). The Australian Administration of Intravenous Medications and Fluids Clinical Policies procedures and guidelines (2017), for example, advise staff ‘perform hand hygiene and don gloves before touching patients’ (p. 8) and when administering IV medications/fluids. In the UK, the East London NHS Foundation Trust (2018) states nurses should ‘put on gloves’ before making up and administering IV medications. The rationale provided is ‘to minimise the risk of contamination and protect against body fluids’ (p. 16). Neither guideline refer to primary research evidence, again underscoring the opportunity for future research in this important area.

Resources on non‐sterile glove use aimed at nursing students are also available. These include a clinical skills video by The University College London on ‘how to prepare intravenous antibiotics for clinical administration’ which advises students, ‘wash your hands and put on gloves’ (UCL Clinical Skills 2019). No evidence was offered to support this educational source. A perfunctory search of nursing texts for students also offers advice with ‘Nursing Interventions & Clinical Skills’ advising students to ‘wear clean gloves when administering parenteral medications’ (Perry et al. 2015, 549). A further skills textbook states nurses are to ‘repeat hand hygiene and apply gloves’ before the administration of IV antibiotics (Biederman et al. 2021, 317). Contemporary nursing textbooks also recommend non‐sterile glove use during IVAB administration (Berman, Snyder, et al. 2021; Berman, Frandsen, et al. 2021; Tollefson and Hillman 2018). These directives presented as standard practice in nursing textbooks are not substantiated by empirical evidence or transparently linked to infection prevention protocols or guidelines. We argue that is concerning given that nursing students should be taught evidence‐based practice so that they are able to deliver safe and effective care. This points to a concerning trend in nursing education where practice norms are translated into procedural knowledge rather than being underpinned by current evidence. This lack of evidence about how to correctly use gloves leaves nursing students struggling to apply correct evidence‐based principles to their practice.

In contrast, some health practitioners and researchers have begun to question the correct use of non‐sterile gloves and have developed educational campaigns to address this issue. This is evident in one of our selected studies: Dunn et al. (2019). Another example is ‘Gloves off! Clean hands. Safe for All’ prepared by the John Hunter Hospital (2023) in New South Wales, Australia, a pilot study to redress the unnecessary use of non‐sterile gloves. While not yet published, the findings of the ‘Gloves off’ pilot project estimate the John Hunter Hospital could potentially save $155,000 (from a 21% reduction in glove use), and reduce waste output by 8 tonnes (John Hunter Hospital 2023).

Implications of incorrect non‐sterile glove use were identified in the included studies in this review. Wilson et al. (2017) found 49% of observed workers practised inappropriate episodes of care, resulting in cross‐contamination between patients, specifically because non‐sterile gloves had not been removed. While non‐sterile gloves provide a barrier reducing the risk of healthcare worker contamination and infection from patients, they also serve as a fomite for cross‐infection if not removed between patients (Alqumber 2021). Dunn et al. (2019) focussed on the financial implications of incorrect non‐sterile glove use. Their study reported the spending on gloves at their study hospital (Great Ormond Street Children's Hospital, London, England) reduced from 289,000 to under 200,000 pounds in the 12 months following their planned intervention to ensure non‐sterile glove use was evidence‐based across all procedures. Dunn et al. (2019) also described this reduction in terms of environmental consequences, reporting a reduction in non‐sterile glove orders by 3.7 million (from 11.1 million to 7.4 million) equating to a reduction in waste from 50 to 32 tonne (30%). The implications for patient health, healthcare costs and the wider environment warrant research dedicated to the subject of non‐sterile glove use in IV antibiotic preparation and administration.

### 4.1 Recommendations for Further Research, Practice and Education

It is evident from the findings of our scoping review that there is a surprising lack of research supporting the use of non‐sterile gloves in the preparation and administration of IV antimicrobials, unless it is a part of standard precautions. This has education, practice and research implications. First, educational bodies and resources need to examine the inclusion of non‐evidence‐based theory and practice in curricula and content. Second, guidelines used in clinical settings must also consider the perpetuation of non–evidence‐based practices in their delivery of care.

Moreover, further research, such as cross‐sectional or observational studies, is urgently needed to investigate clinicians' wearing habits and practices. This will enhance our understanding of non‐sterile glove use and inform the development of guidelines. Finally, there is an imperative to understand the reasoning for this apparently non–evidence‐based practice more broadly, and research into the phenomenon must be considered. Randomised controlled trials can evaluate the effects of glove use on infection and skin outcomes, and qualitative studies can explore healthcare workers' attitudes and decision‐making processes. Combining these methods will provide comprehensive evidence to inform guideline development and improve nursing practice.

### 4.2 Limitations

We acknowledge that no selected articles directly addressed correct non‐sterile glove use during intravenous antimicrobial preparation and administration in clinical practice. Furthermore, the exclusion of non‐English language resources may have influenced the international representation of the findings. This decision was based on practical considerations such as the language capabilities of the research team and resource constraints; however, it may have resulted in the omission of relevant studies from non‐English‐speaking regions. Another issue is that the quality of the three selected papers in terms of rigour, transparency and approach varied, which introduces a potential source of bias.

## 5 Conclusion

This scoping review has revealed a lack of evidence to support or refute the use of non‐sterile gloves in IV antibiotic preparation and administration. It also reveals a lack of evidence based on the wider topic of non‐sterile glove use in nursing practice overall. The confusing and inconsistent messages suggest a lack of evidence‐based practice in this area and do nothing to reduce inappropriate non‐sterile glove use, the consequences of which are significant to patient health, the environment and healthcare costs. This gap challenges current nursing procedures/protocols and educational content, emphasising the urgent need for rigorous research to inform evidence‐based guidelines. Nursing leaders, educators, researchers and policymakers must collaborate to reassess existing practices and protocols, update curricula and promote research that enhances patient safety, optimises resource use and reduces the impact on the environment. Addressing this gap in the existing evidence will empower nurses to deliver safer and more effective care that is grounded in robust evidence.

## Author Contributions

D.M., A.T.‐B., P.Z., N.R.A., S.G.M.A., M.C., C.C. and A.A. were involved in the conception and design of the study and protocol. S.G.M.A., N.R.A., N.Z., M.C., C.C. and A.T.‐B. conducted database searches. P.Z., A.T.‐B., W.L., L.A., C.C. and S.G.M.A. applied inclusion and exclusion criteria to the studies. P.Z., L.A., M.C., S.G.M.A. and A.A. conducted data extraction. P.Z., L.A., N.R.A., M.C. and A.A. appraised the studies. L.A., D.M., P.Z., C.C., A.T.‐B., W.L., S.G.M.A. and N.R.A. were involved with drafting the article. D.M., P.Z., L.A., N.R.A., C.C., M.C., A.T.‐B., S.G.M.A. and A.A. reviewed the article critically for important intellectual content. All authors provided final approval of the version to be submitted.

## Conflicts of Interest

The authors declare no conflicts of interest.

## Supporting information


**Appendix S1:** jan70197‐sup‐0001‐AppendixS1.docx.

## Data Availability

The authors have nothing to report.
